# Quality of care in gout: a clinical audit on treating to the target with urate lowering therapy in real-world gout patients

**DOI:** 10.1007/s00296-017-3777-3

**Published:** 2017-07-26

**Authors:** Carly A. Janssen, Tim L. Th. A. Jansen, Martijn A. H. Oude Voshaar, Harald E. Vonkeman, Mart A. F. J. van de Laar

**Affiliations:** 1 Department of Psychology, Health and Technology, Arthritis Center Twente, University of Twente, PO BOX 217, 7500 AE Enschede, The Netherlands; 2 Department of Rheumatology, Viecuri Medical Center, Venlo, The Netherlands; 3 Department of Rheumatology and Clinical Immunology, Medisch Spectrum Twente, Enschede, The Netherlands

**Keywords:** Gout, Serum urate, Treat-to-target, Hyperuricemia, Care

## Abstract

The current paper aimed to describe the quality of care for gout patients by showing the clinical outcomes achieved in two patient cohorts in which differing targeted urate lowering therapy (ULT) treatment approaches were employed, both aiming to reach the European League Against Rheumatism recommended serum urate (sUA) targets. A retrospective medical chart review study was conducted. Data from the medical records of gout patients from two clinical centers in The Netherlands, both applying targeted ULT treatments (albeit using different approaches), were reviewed. Patients in cohort A were given a combination of xanthine oxidase inhibitors with uricosurics if treatment with allopurinol monotherapy failed to reach sUA target levels, whereas patients in cohort B were treated with sequential monotherapy. Data on patient characteristics and clinical outcomes were collected. A total of 177 patient dossiers were included: 99 from cohort A and 78 from cohort B. The great majority (*n* = 146, 82.5%) of the patients in both cohorts had a current sUA level <360 µmol/L. In addition, more than half (*n* = 104, 58.8%) of the patients met the stringent sUA target level of <300 µmol/L. The largest reductions in mean sUA levels were observed for patients who were treated with combination therapy. This clinical audit of two cohorts of gout patients provides initial—yet promising—results regarding the proportion of real-world gout patients in whom recommended that sUA target levels can be achieved, and demonstrates the added value that a targeted treatment approach may have in reaching these goals.

## Introduction

Gout is one of the most common rheumatic diseases, with a particularly high prevalence in developed countries, where a growing percentage of the population is suffering from obesity and other gout-related comorbid conditions [1]. Various reports have shown the prevalence of gout to be increasing over time, which is frequently attributed to changing lifestyles, the increasing longevity of the population, and the accumulation of gout risk factors in older age [1–3]. Gout is a crystal-induced arthritis that may develop when serum uric acid levels surpass the saturation point for monosodium urate. Typically, gout initially presents as intermittent episodes of inflammation at the first metatarsophalangeal joint, recognized for causing moderate to severe pain, erythema, and joint swelling [4]. If left untreated, a chronic course may develop, characterized by persistent inflammation and visible urate deposits (referred to as tophi), potentially causing bone erosion, irreversible joint damage, and significant disability [5].

The progression or recurrence of disease may be prevented with urate lowering therapy (ULT) that aims to reduce and maintain serum urate (sUA) levels below the saturation point for monosodium urate [6–8]. Although strategies for treating hyperuricemia have proven to be efficacious in clinical trials, the quality of care and management of gout in clinical practice are rarely reported and evidently remain suboptimal as gout patients are reportedly still often misdiagnosed and undertreated [9, 10]. In 2006, the European League Against Rheumatism (EULAR) convened a task force to address these challenges by publishing management guidelines [6, 11]. Recently, these guidelines have been updated to incorporate new developments in the understanding of gout pathophysiology and the availability of novel therapeutic options [12].

Following recommendations for other rheumatic and non-rheumatic conditions, in which treating to a therapeutic target is becoming the standard of care, the 2006 and the updated guidelines for the management of gout recommend lowering and maintaining the sUA <6 mg/dL (360 µmol/L) for all patients on ULT [6, 12]. For patients with severe gout, a target of <5 mg/dL (300 µmol/L) is recommended. However, evidence supporting the added value and feasibility of applying a treat-to-target (TTT) approach in normalizing sUA levels in gout patients remains largely absent and these recommendations are, therefore, currently based on expert opinion alone [13].

In this paper, we describe the quality of care in gout by showing the clinical outcomes achieved in two Dutch patient cohorts in which different targeted treatment approaches were employed; however, both aim to reach the EULAR-recommended sUA targets.

## Methods

### Study sites and patients

A retrospective medical chart review study was conducted among patients suffering from gout, being treated at the rheumatology departments of two rheumatology clinics in The Netherlands, with both clinics using targeted ULT treatment approaches. At the first clinic, the medical records of patients with a clinical diagnosis of gout being followed up for their gout at the outpatient department rheumatology, while using ULT in the maintenance phase (3 months or longer), were included and are referred to as cohort A. At the time of inclusion, none of these patients had pre-terminal renal insufficiency (<10 ml/min) or were under treatment for gout flares with colchicine, prednisolone, or nonsteroidal anti-inflammatory drugs. At the second clinic, the medical records of all patients with a clinical diagnosis of gout were included and are referred to as cohort B. No exclusion criteria were employed. For both cohorts, patient monitoring visits, along with laboratory measurements, had been carried out to meet local standard of care procedures and patient needs.

In cohort A, a ULT-targeted treatment approach was employed allowing combination of xanthine oxidation inhibitors with uricosurics if treatment with allopurinol monotherapy failed to reach the sUA target of <360 µmol/L and ideally <300 µmol/L provided that the patient’s kidney and liver functions, as well as medication tolerance posed no restrictions. In cohort B, a sequential monotherapy approach was followed that aimed for an sUA level <360 µmol/L. In both cohorts, therapy selection, dosages, and switches were made at the discretion of the attending rheumatologist based upon the clinical and biochemical status of the patient and patient experiences.

Data from the patient dossiers were gathered during the period of September to October 2016 and we aimed to retrieve data from approximately 100 consecutive gout patient dossiers from each clinic. Given the nature of the study, and in accordance with the relevant Dutch laws, no approval by the ethical review board was required.

### Data collection and analyses

Data collected from the electronic patient dossiers included patient characteristics (i.e., age, sex, weight, and length), treatment followed, and treatment dosages. Blood measurements were collected, including serum creatinine levels and sUA levels. The latter consisted of two sUA measurements: the maximum sUA level, representing the highest sUA level ever measured in the past, and the current sUA level at the time of data retrieval.

All analyses were performed using SPSS, version 22. Categorical variables were summarized using percentages and compared between groups using Chi-square statistics. Continuous variables were summarized using the mean and standard deviation and compared using Welch’s or paired samples *t* tests, as appropriate. A significance level of *α* = 0.05 was maintained for all analyses. No corrections were applied for multiple comparisons.

## Results

### Patients’ characteristics

In total, 177 patient dossiers were included in the study, of which 99 were from cohort A and 78 from cohort B. In cohort A, all patients and, in cohort B, 62 patients (79%) had crystal-proven gout. No significant differences were observed between the patient groups for age, gender, or body mass index (BMI). In both cohorts, the majority of patients (>80%) were male with a mean (SD) age of 67 (12) and 68 (12) years for cohorts A and B, respectively. Patients in cohort A had a mean (SD) BMI of 30 (5) and in cohort B 30 (6). The mean (SD) serum creatinine in cohort A was 123 (58) µmol/L, which was significantly higher than that of cohort B, namely, 101 (37) µmol/L. The mean (SD) maximum sUA level was significantly higher in patients in cohort A at 588 (115) µmol/L than in cohort B at 545 (74) µmol/L, while for the mean (SD) current sUA level, no significant difference was found between cohorts A and B, at 282 (89) µmol/L and 300 (77) µmol/L, respectively.

### Meeting the sUA targets

**Fig. 1:**
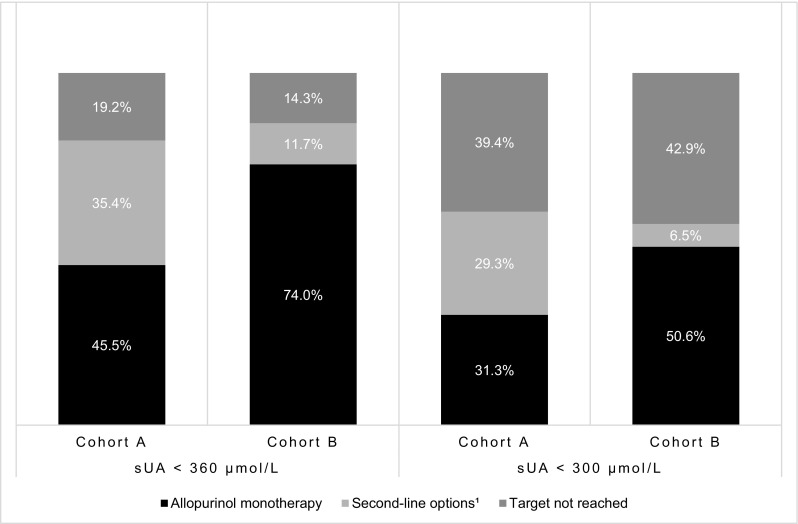
Percentage (%) of gout patients in each cohort meeting the EULAR-recommended serum urate (sUA) target levels of <360 and <300 µmol/L at the time of the study with use of allopurinol monotherapy and second-line treatment options. Febuxostat monotherapy, benzbromarone monotherapy, and combination therapy are together defined as second-line treatment options. ^1^Combination therapy was only applied in cohort A

At the time of the study, 146 out of the 177 patients (82.5%) had a current sUA below the sUA target of 360 µmol/L. More than half of the total sample (*n* = 104, 58.8%) also had a current sUA below the more stringent target of 300 µmol/L. In cohort B, 74.0% (*n* = 57) of the patients had a current sUA below 360 µmol/L when being treated with allopurinol monotherapy at the time of the study. In cohort A, this was 45.5% (*n* = 45). With the use of second-line ULT treatment options, a large proportion of the patients in each independent cohort were able to meet the <360 µmol/L sUA target, with 80 patients (80.8%) in cohort A showing a current sUA below the sUA target and 66 patients (85.7%) in cohort B (Fig. 1). The stringent target of <300 µmol/L was met by 44 patients (57.1%) in cohort B and in 60 patients (60.6%) in cohort A.

### Descriptive results per ULT treatment option

#### Medication use

Table 1 summarizes the ULT medication use at the time of the study, stratified by cohort. In general, patients were treated more aggressively in cohort A. Allopurinol, febuxostat, and benzbromarone monotherapy were all given at higher mean (SD) dosages (milligram) to cohort A [373 (169); 80 (15); 75 (29), respectively] than to cohort B [302 (116); 53 (21); 60 (22)].

**Table 1:** Total number (*N*) and percentage (%) of gout patients in cohort A and cohort B receiving urate lowering therapy (ULT) grouped per ULT type, including the maximum and current mean serum urate (sUA) levels and sUA targets met within each ULT treatment group

Type of ULT treatment	*N* (%^a^)	Mean (SD) sUA µmol/L maximum	Mean (SD) sUA µmol/L current	sUA <360 µmol/L, *N* (%)	sUA <300 µmol/L, *N* (%)
Cohort A					
Allopurinol monotherapy	58 (58.6)	558 (94)	300 (86)	45 (77.6)	31 (53.4)
Febuxostat monotherapy	16 **(**16.2)	598 (99)	277 (85)	14 (87.5)	11 (68.8)
Benzbromarone monotherapy	4 (4.0)	608 (131)	330 (65)	2 (50.0)	1 (25.0)
Allopurinol/benzbromarone combination therapy^b^	15 (15.2)	612 (120)	213 (82)	14 (93.3)	13 (86.7)
Febuxostat/benzbromarone combination therapy^b^	6 (6.1)	793 (163)	255 (91)	5 (83.3)	4 (66.7)
Cohort B					
Allopurinol monotherapy	66 (84.6)	540 (70)	296 (70)	57 (86.4)	39 (59.1)
Febuxostat monotherapy	6 (7.7)	592 (111)	380 (90)	4 (66.7)	1 (16.7)
Benzbromarone monotherapy	5 (6.4)	554 (81)	234 (58)	5 (100.0)	4 (80.0)
No ULT	1 (1.3)	NA	NA	NA	NA

Total sample size of cohort A = 99 patients and cohort B = 78 patients

*ULT* urate lowering therapy, *NA* not applicable, *SD* standard deviation, *sUA* serum urate

^a^ Sum of percentages within each cohort is equal to 100

^b^ Combination therapy was only given in cohort A

#### sUA levels

The second part of Table 1 summarizes the mean (SD) sUA maximum and sUA current per ULT treatment group, along with the percentages of patients within each group achieving sUA target levels. The subpopulations of patients treated with second-line monotherapy (including both febuxostat and benzbromarone monotherapy) or combination therapy had a significantly higher mean (SD) maximum sUA compared with patients treated with allopurinol monotherapy at the time of the study, 591 (99) µmol/L, 658 (150) µmol/L, and 549 (82) µmol/L, respectively. Patients treated with combination therapies also achieved a substantially greater mean (SD) decrease in sUA levels compared to other patient treatment groups [432 (152) µmol/L versus 294 (115) µmol/L for second-line monotherapy and 248 (106) µmol/L for allopurinol monotherapy].

## Discussion

In this paper, we report the result of a clinical audit on the quality of care in gout using a retrospective chart review study in two independent cohorts in which gout patients were treated according to different ULT approaches, both aiming to achieve sUA target levels as recommended in the updated EULAR gout guidelines. The results presented here illustrate the sUA levels and clinical outcomes that may be reached in everyday clinical practice using currently available ULT and a targeted treatment approach. An analysis of our quality of care (the combination of prescribed treatment, instructing, and monitoring of gout patients) is of importance to get a sense of where opportunities may lie for further improvement.

Based on our data, we postulate that achieving EULAR-recommended sUA levels seems to be a realistic goal in clinical practice for gout patients, using currently available ULT, patient education, and monitoring of sUA-targeted treatment. The great majority (82.5%) of the gout patients in the studied cohorts had a current sUA <360 µmol/L. This is in contrast to other studies in which much lower proportions of patients were able to meet sUA levels <360 µmol/L, ranging from 21 to 77% [14–19]. In those studies, failure to reach sUA targets was seen as multifactorial, but mainly attributed to inadequate dose titration of ULT according to sUA measurements, as well as to infrequent patient monitoring and treatment incompliance. In our study, patient monitoring visits had been made according to daily routine practice and patient needs, in principle, allowing for ULT dosages and treatments to be adjusted according to sUA measurements, which might explain the higher percentages of patients meeting sUA targets in our study. Since both data on the frequency of patient monitoring visits and patient follow-up data were beyond the scope of this clinical audit, the extent to which dose and medication adjustments were actually steered in response to patients’ current sUA levels could not be assessed. Moreover, between the rather similar cohorts, significant variation existed among the percentages of patients treated with allopurinol monotherapy and having a current sUA below 360 µmol/L. This could have occurred, because a more stringent target of <300 µmol/L was striven for in some patients in cohort A. However, physician-dependent or unobserved patient-related factors might also have contributed to changes in treatment options or dosage adjustments. Nonetheless, our study shows that most patients can achieve sUA target levels if medication is titrated to reach predefined sUA targets, providing a proof-of-concept of the feasibility of a TTT approach in gout.

In a TTT approach, patients typically follow a therapeutic process in which failing to respond to a treatment option leads to switching to other, perhaps more costly, treatment options to achieve prespecified sUA target values. In the current study, 31 gout patients (17.5%) failed to have a current sUA below 360 µmol/L of whom eight patients (4.5%) were already using the second-line options of monotherapy or combination therapy. Therefore, these patients could benefit from alternative therapy choices. One such treatment for patients with severe debilitating, chronic tophaceous gout, who have exhausted other treatment options, is pegloticase. Other therapies, such as the uricosuric lesinurad, might also become available for this patient group in the near future. The added value for the quality of care of gout patients of novel treatment options should be assessed in future real-life studies.

There are a few limitations to this study. First, only gout patients undergoing follow-up at rheumatology departments were included in this study, probably leading to a sample of patients with severe or complex gout. Since in The Netherlands, the majority of gout patients are treated by their general practitioner, future studies in primary care settings seem warranted. Second, data relating to patient-reported outcomes, other gout outcomes (e.g. number of flares), treatment costs, and drug safety were not available for this study. Although previous studies have demonstrated considerable correlation between sUA and many of these, future prospective and clinical studies should include such outcome domains to better characterize potential differences between treatment strategies [5, 19, 20]. Strengths of the current study are the use of real-life data and the employment of few exclusion criteria, therefore, reflecting the experience of gout patients seen in daily clinical practice.

In summary, the findings of this study provide an initial proof-of-concept of the favorable outcomes in terms of the percentage of real-world gout patients for whom recommended sUA target levels can be achieved when using ULT, and the added value a targeted treatment approach may have in reaching these goals. To evaluate potential differences between treatment strategies regarding efficacy, safety, costs, patient-reported outcomes, and the impact on society, prospective, pragmatic studies are needed to further investigate the TTT principle of sUA levels in gout patients.
